# Systematic review of surface disinfection: Spraying versus wiping for COVID-19 prevention

**DOI:** 10.4102/jphia.v16i2.597

**Published:** 2025-01-28

**Authors:** Babasola O. Okusanya, Muzzammil Gadanya, Anthony Nlemadim, Victoria Adaramoye, David O. Akeju, John Ehiri, Martin M. Meremiku

**Affiliations:** 1 Department of Obstetrics and Gynaecology, Faculty of Clinical Sciences, College of Medicine, University of Lagos, Idi-Araba, Lagos, Nigeria; 2 Nigeria Centre for Disease Control, Abuja, Nigeria; 3 Department of Paediatrics, University of Calabar Teaching Hospital, Calabar, Nigeria; 4 Department of Obstetrics and Gynaecology, Lagos University Teaching Hospital, Lagos, Nigeria; 5 Department of Sociology, Faculty of Social Sciences, University of Lagos, Lagos, Nigeria; 6 Department of Health Promotion and Sciences, College of Public Health, University of Arizona, Tucson, United States of America

**Keywords:** surface disinfection, community disinfection, material disinfection, decontamination, disinfection

## Abstract

**Background:**

Within countries, community spread of severe acute respiratory syndrome coronavirus-2 (SARS-CoV-2) propagated the infection despite the use of non-pharmaceutical interventions.

**Aim:**

To evaluate the effectiveness of disinfecting surfaces and materials in the community by spraying compared with wiping (mechanical cleaning) or nothing for SARS-CoV-2 infection prevention.

**Setting:**

This research was conducted in a global context.

**Method:**

We searched six databases for eligible studies from 01 January 2020 to 06 September 2022. Spraying disinfectants was the intervention, while wiping or nothing was the comparison. Review outcomes include SARS-CoV-2 infection, the incidence of adverse effects and operator satisfaction. The review was registered on Prospero: CRD42022356276.

**Results:**

We found no studies that compared spraying with wiping or had human participants. Three studies with indirect evidence, published between 2021 and 2022 in Japan, South Korea and Spain, were included. Dry fog spraying of 8 700 parts per million (ppm) of hypochlorous acid solution or 56 400 ppm of hydrogen peroxide solution reduced the infectious viral titre. Wiping with 1000 ppm of sodium hypochlorite for 1 min completely reduces SARS-CoV-2 viruses on stainless steel. Also, wiping with 500 ppm of bleach for 5 min completely reduces the virus on kraft paper and polypropylene. No viruses were detected on any surface after wiping with 1000 ppm of bleach for 5 min.

**Conclusion:**

This review provides basic scientific evidence that either spraying disinfectants as dry fog or wiping has some disinfectant effects on surfaces and materials.

**Contribution:**

Although the review included no human studies, both methods of disinfection can be practiced in the community for SARS-CoV-2 infection prevention.

## Background

The community, including households’ items such as tables and chairs, water dispensers and television (TV) remotes, dining tables and bedsheets, car doorknobs, car steering wheels and seats of infected persons have all been reported to be contaminated within 3 days of severe acute respiratory syndrome coronavirus-2 (SARS-CoV-2) infection diagnosis.^1^ In the community, human-to-human SARS-CoV-2 infection is the main reservoir for the prolongation of the pandemic despite the combined use of vaccination and non-pharmaceutical interventions (NPIs).^2^ It is, therefore, important to prioritise identifying modalities for reducing SARS-CoV-2 infection in households and communities, including spray disinfection.

Spraying surfaces with virucidal agents is one method of community and household disinfection. Spraying alcohol on wet wipes was effective in preventing SARS-CoV-2 infection in schools.^3^ More so, ethanol (EtOH) at 40% dilution and bleach at 1:2000 dilution have been reported as effective household cleaning agents for SARS-CoV-2 infection prevention.^4,5^ Spraying disinfectants reported to reduce SARS-CoV-2 infection on surfaces accessible to the public and within households might be an effective intervention for the prevention of community infection, especially when used at scale with other NPIs.

There are bleach and alcohol sprays that are used for surface disinfection for SARS-CoV-2 infection prevention. Sodium hypochlorite solution, which is bleach, has a general chemical reaction against organic compounds, which irreversibly denatures proteins, thereby giving them broad effectiveness. Similarly, alcohols have broad-spectrum activities against viruses, causing membrane damage and protein denaturation.^6^ Alcohol (EtOH) is most effective at 60% – 90% concentration because it relies on water molecules for optimal virucidal activities.^6^

Wiping surfaces with soap and water or virucidal agents such as bleach and alcohol is one method of community and household disinfection. When wiping, the disinfectant may be applied directly to the contaminated surface, which is spread over with another substance, such as a napkin. Alternatively, there are readymade wipes pre-saturated with disinfectants. Wiping, unlike spraying, has a mechanical component to its disinfection process. During wiping, the disinfectant is spread and rubbed over the surface. This not only disinfects the surface or material but also dislodges any particle on the surface or material and, therefore, it might be more effective than spraying.^7^ However, wiping increases the likelihood of disinfectant contact with a person’s skin, unlike spraying. Therefore, wiping might produce better decontamination of surfaces and materials in the community for SARS-CoV-2 infection prevention yet increase the likelihood of adverse effects.

Evaluating the effectiveness of surface and materials disinfection with sprays in community settings is important. In low- to middle-income countries (LMICs), people reported interactions within the community, including visits to markets, families and friends during the SARS-CoV-2 infection lockdown.^8^ Low- to middle-income countries have large family sizes with a high frequency of contacts within the household and a high frequency of contacts has been reported as a determinant for SARS-CoV-2 infection propagation.^9^ A systematic review reported limited effectiveness of a single NPI, while a combination of multiple NPIs was more effective.^10^ There were also conflicting recommendations for face covering use for SARS-CoV-2 infection prevention.^11,12^

The objective of this systematic review was to assess the effectiveness of the disinfection of surfaces and materials by spraying compared with wiping (mechanical cleaning) for SARS-CoV-2 infection prevention.

## Methods

### Search strategy

We searched the following databases: The Cochrane Library – Central Register of Controlled Trials (CENTRAL) and Cochrane Database of Systematic Review; PubMed, EMBASE, EPOC (The Effective Practice and Organization of Care) and Latin America and the Caribbean Literature on Health Sciences (LILACS) for the period January 2020 to 06 September 2022 (see Online Appendix 1 for detailed search strategies). We checked the reference lists of retrieved studies for additional reports of relevant studies. There were no language restrictions. We used PRISMA (Preferred Reporting Items for Systematic Reviews and Meta-Analyses) guidelines and flow diagrams to report the search and selection of studies.

### Inclusion and exclusion criteria

In order to ensure a broad and comprehensive overview of this research interest, we developed our inclusion and exclusion criteria.

#### Types of studies

We considered randomised controlled studies for inclusion, and in their absence we considered cohort studies (prospective or retrospective), case-control studies, controlled before and after studies (CBA), interrupted time series, systematic reviews (randomised control trial [RCT] and non-RCT studies), observational studies, ecological studies, clinical reports, outbreak reports and non-predictive modelling.

#### Types of participants

These are people in the community, including the workplace, public transport system and households.

#### Intervention

Spraying of disinfectants, including bleach and alcohol sprays.

### Comparisons

- Wiping (mechanical cleaning) of surfaces and materials for SARS-CoV-2 infection prevention.
- Nothing (No disinfection).

### Outcome measures

The outcome measures are:

- Spread of SARS-CoV-2 infection.
- Incidence of adverse effects for example airway irritation, disinfectant poisoning or any other reported adverse effect.
- Satisfaction with either spraying or wiping (mechanical cleaning) with disinfectants of surfaces and materials.

### Selection of studies

We used a Microsoft Excel sheet for screening the search outputs. Two review authors in two pairs (V.A. and A.N.; M.G. and D.A.) independently screened the literature search results for potentially relevant studies and obtained the full reports of potentially relevant studies for further assessment. They independently applied the inclusion criteria to the full-text reports using an eligibility form and scrutinised publications to ensure each study was included in the review only once. We resolved disagreements through a consensus within the review team. We listed excluded studies and the reasons for their exclusion.

### Data extraction and management

We used a Microsoft Excel sheet for data extraction. We extracted data related to place, research method, year of publication, authors, interventions and outcomes for all included studies where available. Two authors (V.A. and D.A.) independently extracted data using a specifically developed piloted data extraction Excel sheet. We resolved disagreements through discussion between all review authors. There were no missing data; therefore, we did not contact the corresponding publication authors. The included studies did not involve human participants and did not report on any of the pre-specified outcomes. In a future update of this review, if we find studies involving human participants, we will extract the number of randomised study or included in the study and the number analysed in each arm or group. For dichotomous outcomes, we will record the number of participants experiencing the event and the number assessed in each arm or group.

### Assessment of risk of bias

Two (A.N. and M.G.) review authors independently assessed the risk of bias in each included study using a ‘Risk of bias’ template of the Cochrane Risk of Bias in Non-randomized Studies – of Interventions (ROBINS-I).^13^ Because included studies involved the use of surfaces in the laboratory and public buses contaminated with SARS-CoV-2 and subsequent disinfection, the ROBINS-I tool was applied with consideration of these peculiarities. Each study was assessed in the following domains: bias because of confounding, bias in the selection of participants into the study, bias in classification of interventions, bias as a result of deviations from intended interventions, bias because of missing data, bias because of measurement of outcomes, and bias because of selection of reported outcomes. In a future update, if we include studies with human participants, we will assess the balance between comparator groups at baseline with respect to the main prognostic or confounding factors. An effort will be made to identify and extract data on potentially confounding variables. We will assess whether the study authors have employed methods to control for selection bias at the design stage (e.g. matching or restriction to subgroups) and their analysis methods (e.g., the use of stratification or regression modelling). For studies with a separate control group (RCTs, non-RCTs, controlled before-after studies), we will assess eight components: generation of the randomisation sequence; allocation concealment; blinding (performance and detection bias); baseline outcome measurement; similarity in baseline characteristics; incomplete outcome data; selective outcome reporting; and other biases.

### Data synthesis and assessment of quality of evidence

We did not find studies homogenous enough to conduct a meta-analysis. Hence, we used the Synthesis Without Meta-analysis (SWiM) reporting guidelines to report the findings of the review. PROSPERO registration: CRD42022356276.

### Ethical considerations

This article followed all ethical standards for research without direct contact with human or animal subjects.

## Results

**FIGURE 1:**
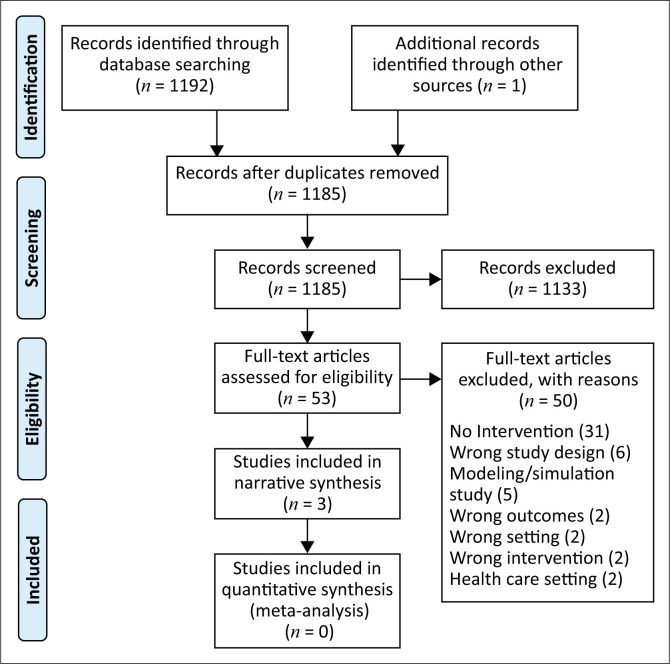
Study selection process.

The search of the databases yielded 1192 titles, and 2 additional titles were identified from a reference list. After the deduplication of titles, 1186 titles and abstracts were screened for eligibility, after which 53 made it to full-text article review. Figure 1 shows the PRISMA flow diagram with the detailed study selection process. We found no studies that compared spraying with wiping, involving humans for inclusion. However, three studies that provided indirect evidence were included. We excluded 50 studies with reasons. Table 1 shows the characteristics of the included studies. The reasons for excluding studies may be found in Online Appendix 2.

**TABLE 1:** Characteristics of included studies.

Study details	Description
**Urushidani 2021** ^14^	
Methods	Controlled laboratory experiment.
Participants	No human participants; involved preparation of hypochlorous acid solution and hydrogen peroxide as disinfectants for spraying as dry fog.
Interventions	Three types of solutions were prepared using different concentrations; (1) ‘Commercially available, weakly acidic (pH 6.5) hypochlorous acid solution with a free available chlorine (FAC) concentration (the total of HOCl and OCl-concentrations) of 250 ppm (Super Jiasui; HSP Corporation, Okayama, Japan) and a solution diluted by distilled water with a FAC concentration of 125 ppm were used’. (2) ‘A solution with a FAC concentration of 8700 ppm, sodium hypochlorite (Hayashi Pure Chemical Ind., Ltd., Osaka, Japan) was dissolved in distilled water, followed by an adjustment of the pH of the solution to 6.5 with HCl (Hayashi Pure Chemical Ind., Ltd.)’. and (3) ‘Commercially available hydrogen peroxide solution (56 400 ppm; Part 1, Decon7; Decon7 Systems LLC., Texas, United States) and solutions diluted by distilled water with hydrogen peroxide concentrations of 11 280, 5640, 2820, and 1410 ppm were prepared. Regarding the negative control, distilled water was sprayed in the form of dry fog’. ‘A closed test chamber of 500 × 700 × 300 mm size, made of acrylic was prepared to fill the space with dry fog. It was set in a biosafety cabinet. A sliding door was set on the front of the chamber for the handling of samples inside the chamber. A 130 sprayer equipped with an impinging-jet atomising nozzle (AE-1 [03C], AKIMist® “E”; H. Ikeuchi & Co., Ltd., Osaka, Japan) was used to spray a disinfectant into the space. To generate aerosolised disinfectants in the form of dry fog, 0.3 MPa compressed air was supplied to the sprayer from a compressor (0.2LE-8SB0; Hitachi Industrial Equipment Systems Co., Ltd., Tokyo, Japan). The spray capacity was 2.3 L per hour and the Sauter mean droplet diameter was 7.5 μm. In the virus inactivation experiment using dry fog spraying, four 90-mm Petri dishes containing 20 mL of distilled water, a thermo-hygrometer, and a 96-well microplate containing dried viral samples were placed in the chamber. Spatial spraying was conducted as follows. A disinfectant was sprayed for 5 seconds at the start of the experiment and left to stand for 4 minutes. Spraying was then repeated 3 more times for 2.5 seconds each, and left to stand for 4 minutes after each spraying. Spraying was performed 4 times, namely, 0, 4, 8, and 12 minutes after the initiation of the experiment, and the total experimental period was 16 minutes’.
Outcomes	Inactivation of SARS-CoV-2 viral titre (1.2 × Log_10_ TCID 50/mL in 5 μL) by disinfectants sprayed as dry fog.
Identification	Country: Japan Authors name: Urushidani Masahiro Email: mkameoka@port.kobe-u.ac.jp
**Jung 2023** ^15^	Controlled laboratory experiment.
Methods	Controlled laboratory simulation ‘Before and After’ study.
Participants	No human participants.
Interventions	Kraft paper, parchment paper, and low-density polyethylene (LDPE) were made into a carrier with a diameter of 8 mm using a punch. Stainless steel (SS), glass, and polypropylene (PP) were selected as the most frequently used food contact surfaces in food processing. SS, glass, and PP were processed to a thickness of 1 mm and a diameter of 1 cm. All surfaces except LDPE were sterilised by autoclave, and LDPE was degreased with acetone and then sterilised by immersion in 70% EtOH and dried aseptically in a class II biosafety cabinet (BSCII). Each surface was inoculated with 10 μL of 6 log TCID 50/mL of the viruses and dried for 1 h in BSCII. The wiping test to verify the interim guidelines was performed on three hard surfaces (SS, glass, and PP). Each carrier was contaminated with the virus under the same conditions as described for the quantitative carrier test. A sterile cotton swab moistened with 70% EtOH, 500 or 1000 ppm NaClO was used to wipe the virus-contaminated hard surface 1–3 times until the dry stains disappeared. Immediately after exposure to the disinfectant for a specified time, the carrier was transferred to 1 mL of DMEM containing 10% FBS to neutralise the disinfectant. All test substances were diluted with hard water and the same test was performed with a sterile cotton swab moistened with hard water to evaluate the effect of the wiping action itself. Virus elution and infectivity evaluation were performed using the same method as described for the quantitative carrier test.
Outcomes	Viability of coronaviruses including two types of SARS-CoV-2 and human coronavirus 229E (229E) at three different temperatures (20 °C, 4 °C, and – 20 °C) on food contact surfaces. Decontamination of surfaces using common disinfectants and UV-C.
Identification	Country: Republic of Korea Authors name: Soontag Jung Email: cchoi@cau.ac.kr
**Moreno 2021** ^16^	
Methods	Controlled ‘Before and After’ study.
Participants	No human participants; Eight (8) polyester swab samples from public buses in Barcelona, Spain.
Interventions	The sampling of the buses for the possible presence of SARS-CoV-2 took place between 20:00 and 03:00 on the night of May 25–26, 2020 in one of the four main bus depots in Barcelona. For the sampling performed before the nightly maintenance and cleaning, polyester swabs were wiped across the left side of call buttons (around 10 cm^2^) and plastic/aluminium holding bars (250 cm^2^, 20 cm above and below each call button). The disinfection was carried out using bleach (manual cleaning with 5% sodium hypochlorite). After each bus had been cleaned and prepared, the sampling procedure was repeated but on the right side of the same call buttons and bars.
Outcomes	Virus detection on surfaces was carried out by analysing the presence of viral RNA with Real-Time Reverse-Transcription PCR (Real-Time RT-qPCR) using three target gene regions: two targets from the RNA-dependent RNA polymerase.
Identification	Country: Spain Authors name: Teresa Moreno Email: teresa.moreno@idaea.csic.es

Note: Please see the full reference list of the article Okusanya BO, Gadanya M, Nlemadim A, et al. Systematic review of surface disinfection: Spraying versus wiping for COVID-19 prevention. J Public Health Africa. 2025;16(2), a597. https://doi.org/10.4102/jphia.v16i2.597, for more information.

HOCl, hypochlorus acid; TCID, tissue culture infectious dose; MPa, megapascals; NaClO, sodium hypoclorite; OCI, hypochlorite; ppm, parts per million; SARS-CoV-2, severe acute respiratory syndrome coronavirus-2; EtOH, ethanol; DMEM, Dulbecco’s Modified Eagle Medium; FBS, Fetal Bovine Serum; UV-C, ultraviolet-C; RNA, bonucleic acid; PCR, polymerase chain reaction.

Included studies were published between 2021 and 2022, in Japan, South Korea and Spain.^14,15,16^ Urushidani et al. in Japan, assessed the inactivation of the SARS-CoV-2 by disinfectants sprayed as dry fog using different concentrations of hypochlorous acid and hydrogen peroxide in a closed chamber inside a level II biosafety laboratory.^14^ Jung et al., working in a Level III biosafety laboratory in the Republic of Korea, inoculated cut surfaces of common food packages such as stainless steel, glass and polypropylene with a live virus.^15^ The surfaces were then swabbed with ethyl alcohol and bleach swabs, and the presence of the virus on the surfaces was checked on the surfaces.^15^ In Barcelona, Spain, buses used for public transportation were assessed for the presence of SARS-CoV-2 before routine maintenance and cleaning at the end of the day. Polyester swabs were wiped across call buttons and plastic and/or aluminium holding bars in the buses before manual cleaning with 5% sodium hypochlorite (bleach).^16^ Then, the sampling procedure was repeated after cleaning. (see Table 1 for details)

Accounting for study design, using the ROBINS-I risk of bias tool, the included studies were assessed to be at low risk of bias. See Table 2 for the risk in each of the domains of ROBINS-I. There was methodical heterogeneity in the included studies. While the study that evaluated spraying disinfectants as dry fog used a chamber inside a biosafety laboratory,^14^ the two studies that evaluated wiping took samples before and after wiping from surfaces of common food packs in biosafety chamber^15^ and from commonly touched surfaces in public buses.^16^

**TABLE 2:** Risk of bias in included studies.

Study ID	Bias because of confounding	Bias in selection of participants into the study	Bias in classification of interventions	Bias because of deviations from intended interventions	Bias because of missing data?	Bias in measurement of outcomes	Bias in selection of the reported result	Overall bias
Urushidani 2021^14^	Low	Low	Low	Low	Low	Low	Low	Low
Jung 2022^15^	Low	Low	Low	Low	Low	Low	Low	Low
Moreno 2021^16^	Low	Low	Low	Low	Low	Low	Low	Low

Note: Please see the full reference list of the article Okusanya BO, Gadanya M, Nlemadim A, et al. Systematic review of surface disinfection: Spraying versus wiping for COVID-19 prevention. J Public Health Africa. 2025;16(2), a597. https://doi.org/10.4102/jphia.v16i2.597, for more information.

### Comparison 1: Spraying versus wiping

We did not identify any study that compared spraying with a wiping method of disinfection for COVID-19 infection prevention. Therefore, we compared spraying with ‘nothing’ and wiping with ‘nothing’.

### Comparison 2: Spraying versus nothing

The review identified a study that compared different concentrations of hypochlorous acid and hydrogen peroxide with distilled water sprayed as dry fog as a disinfectant for SARS-CoV-2.^14^ The study did not have human participants and did not report any of the outcomes pre-specified for this review. At 250 parts per million (ppm) hypochlorous acid solution, the viral titre (1.2 × Log_10_ tissue culture infectious dose [TCID]_50_/mL in 5µL) of SARS-CoV-2 was not reduced. However, after about 16 min, 8700 ppm hypochlorous acid solution or 56 400 ppm hydrogen peroxide solution sprayed as dry fog significantly reduced (*p* < 0.0001) the infectious titre of SARS-CoV-2.^14^ Compared to hypochlorous acid or hydrogen peroxide, dry fog spraying of distilled water did not reduce the viral infectivity of SARS-CoV-2.^14^

### Comparison 3: Wiping versus nothing

Two included studies did not compare wiping with any method. The studies did not have human participants and did not report any of the outcomes pre-specified for this review.

Jung et al. demonstrated in the laboratory that wiping with 1000 ppm of sodium hypochlorite (bleach) for 1 min completely reduces SARS-CoV-2 viruses on stainless steel.^15^

Wiping with 500 ppm of bleach for 5 min completely reduces the virus on kraft paper and polypropylene, with a decrease of > 3 log on glass.^15^ Wiping with 1000 ppm sodium hypochlorite for 1 min showed a complete reduction in viruses on stainless steel only and > 3 log reductions on parchment paper, glass and polypropylene.

At 1000 ppm for 5 min, no viruses were detected on any surface, although trace amounts of two SARS-CoV-2 strains were present on low-density polyethylene (LDPE) (0.55 TCID 50/mL for S and L types).^15^

Ethyl alcohol effectively reduced the infectivity of six surfaces contaminated with SARS-CoV-2, and it was not detected on kraft paper, stainless steel and glass when wiped with 50% and 70% ethyl alcohol concentrations (SARS-CoV-2 L and SARS-CoV-2 S were reduced by 2.98 ± _0.13 and 2.85 ± _0.08 log TCID 50/mL at 50% and 3.08 ± _0.06 and 3.10 ± _0.03 log TCID 50/mL at 70%, respectively) for 1 min.^15^ For complete reduction, surfaces require wiping with 1000 ppm NaClO or 70% EtOH for at least 5 min or the use of 500 ppm NaClO for 10 min.

However, in the study that detected the presence of SARS-CoV-2 in public buses in Spain, eight surfaces were wiped (manually cleaned) with bleach.^16^ Of these surfaces, four (50%) had SARS-CoV-2 detected before wiping. After wiping with bleach, no surfaces had SARS-CoV-2 detected on it.^16^

When the infectivity of coronavirus was assessed at a different temperature, SARS-CoV-2 rapidly decreased irrespective of the surface type at 20 °C (decreased by > 1.5 log TCID 50/mL after 2 h on kraft paper and parchment paper, decrease by > 1.5 log after 8 h on LDPE).^15^ At 4 °C, the infectivity of SARS-CoV-2 was variable; depending on the surface type (decreased by > 3 log TCID 50/mL after 24 h on kraft paper, decreased by > 3 log TCID 50/mL after 72 h on parchment paper, decreased by > 3 log after 120 h on LDPE).^15^ However, at –20 °C, the infectivity of SARS-CoV-2 hardly reduced (decreased by > 1 log TCID 50/mL after 120 h but hardly reduced on the other two surfaces).^15^

## Discussion

The objective of the systematic review was to evaluate the effect of disinfecting surfaces and materials within the community, households, workplaces and public transport systems with spraying compared with wiping (mechanical cleaning) for SARS-CoV-2 infection prevention. The main finding of the review was that we did not identify any studies comparing spraying with wiping involving human participants for inclusion. Therefore, the main outcomes set *a priori*, including SARS-CoV-2 infection, the incidence of adverse effects, and satisfaction with spraying or wiping surfaces and materials with disinfectants, could not be evaluated.

Laboratory simulation and/or modelling techniques and sampling of surfaces commonly touched in public buses indicate that spraying or wiping surfaces with disinfectants has some effect on community SARS-CoV-2 infection prevention.^14,15,16^ Spraying or wiping (mechanical cleaning) to inactivate SARS-CoV-2 is influenced by the disinfectant concentration, the wiping duration and the surface being disinfected. For instance, it takes about 16 min of applying 8700 ppm hypochlorous acid solution or 56 400 ppm hydrogen peroxide solution sprayed as dry fog to achieve a significant reduction (*p* < 0.0001) of infectious titre of SARS-CoV-2.^14^ Also, after 1 min of wiping, 1 000 ppm of hypochlorous acid disinfected SARS-CoV-2 on stainless steel but not on kraft paper and polypropylene.^15^ However, at 1 000 ppm, hypochlorous acid inactivated SARS-CoV-2 on all surfaces after 5 min of wiping.^15^ Similarly, using 50% or 70% of ethyl alcohol to wipe surfaces for a minute inactivated SARS-CoV-2 on all surfaces.^15^ The study that mimics real-life situations used 5% hypochlorous acid to disinfect commonly touched surfaces (call buttons and holding bars) in public buses and demonstrated the effectiveness of the wiping method disinfection.^16^

Identified studies had no human participants, although they reported ‘basic science’ evidence on the effects of spraying or wiping under controlled laboratory conditions. Because the evidence does not reflect real-life situations when people encounter surfaces within households, workplaces and the community, the evidence should be taken with caution. More so, wiping was performed on surfaces with small sizes and a limited number of surfaces and materials, which were of different materials involved. For instance, the concentration of disinfectants and duration of wiping of different surfaces were not the same. This greatly influences the inactivation of SARS-CoV-2 on surfaces.

In another systematic review of chlorine-based surface disinfectants, application methods, soil load and surface types influenced the effect of chlorine-based surface disinfectants.^17^ Similarly, cleaning public bus call buttons and holding bars was performed for 2 h – 5 h at the end of the day,^16^ which is not pragmatic for commonly touched surfaces in households, public transport and the workplace. This cannot be equated to wiping disinfectants over commonly touched surfaces during or between public buses’ use, especially as very few (four) samples were tested after cleaning and disinfection.

### Implications for public health practice

The purpose of the review was to identify which method of disinfectant application, between spraying and wiping, was effective. The studies included did not directly compare the two methods. Also, the included studies had no human participants and were largely held in a laboratory-controlled environment. This made it impossible to evaluate the effect of either method of disinfectant application for SARS-CoV-2 disinfection of surfaces and materials in the community. Therefore, the use of either spraying or wiping methods of disinfectant application for SARS-CoV-2 should be continued as recommended by the manufacturers of the disinfectants.

### Implications for future research

This review has highlighted the unavailability of well-conducted human studies that evaluated the effectiveness of spraying and wiping methods of disinfectant application for community prevention of SARS-CoV-2. The basic science evidence from the included studies has provided some foundation for well-developed observational studies to be conducted. While it might be unethical to conduct experimental studies, observational studies, including controlled ‘Before and After’ studies and cohort studies, might be conducted. Such studies should be designed to assess the adverse effects of common disinfectants used either by spraying or wiping methods. This is necessary as there was an increase in reported cases of adverse reactions to disinfectants reported to poison control centres during the pandemic.^18,19^ It will also be good to evaluate people’s preferences between spraying and wiping methods of disinfectant application.

### Strength and limitations

The strength of the review is the meticulous search of the databases for eligible studies on community disinfection for SARS-CoV-2 prevention using spraying or wiping methods. The restriction of the search from January 2020 to September 2022 ensured that studies conducted after the COVID-19 pandemic began were eligible for inclusion. Also, the search was comprehensive enough to identify any eligible studies because no language restrictions were applied. That two review authors independently screened the titles identified from the database search for inclusion and performed the risk of bias assessment were strengths. Also, the ROBINS-I risk of bias assessment tool was used to assess the risk of bias in included studies with due consideration of the laboratory design of the included studies.

This strength did not preclude limitations in the review. The most important of which is the indirect application of the evidence to human activities in the community, including households, workplaces, and public transport systems. This is because we found no studies involving humans or any that reported the pre-specified review outcomes. Also, there was a sparse sampling of surfaces and materials after disinfection.

## Conclusion

This systematic review found no studies that compared spaying and wiping for SARS-CoV-2 community disinfection of surfaces and materials. Indirect evidence from laboratory simulation or modelling studies indicates that the effects of spraying or wiping disinfectants are influenced by the concentration of the disinfectants, duration of use, and the type of surface or materials being disinfected.
